# A Motivational-Developmental Free Response Assessment Through a Bifactor Lens

**DOI:** 10.3389/fpsyg.2021.770327

**Published:** 2021-12-03

**Authors:** David Alpizar, Brian F. French

**Affiliations:** Learning and Performance Research Center, Washington State University, Pullman, WA, United States

**Keywords:** local item dependence, restricted bifactor, testlet, method effect, factor analysis

## Abstract

The Motivational-Developmental Assessment (MDA) measures a university student’s motivational and developmental attributes by utilizing overlapping constructs measured across four writing prompts. The MDA’s format may lead to the violation of the local item independence (LII) assumption for unidimensional item response theory (IRT) scoring models, or the uncorrelated errors assumption for scoring models in classical test theory (CTT) due to the measurement of overlapping constructs within a prompt. This assumption violation is known as a testlet effect, which can be viewed as a method effect. The application of a unidimensional IRT or CTT model to score the MDA can result in imprecise parameter estimates when this effect is ignored. To control for this effect in the MDA responses, we first examined the presence of local dependence via a restricted bifactor model and Yen’s *Q3* statistic. Second, we applied bifactor models to account for the testlet effect in the responses, as this effect is modeled as an additional latent variable in a factor model. Results support the presence of local dependence in two of the four MDA prompts, and the use of the restricted bifactor model to account for the testlet effect in the responses. Modeling the testlet effect through the restricted bifactor model supports a scoring inference in a validation argument framework. Implications are discussed.

## A Motivational-Developmental Free Response Assessment Through a Bifactor Lens

Student’s motivational and developmental (MD) attributes are vital for student adjustment, learning outcomes, achievement, and retention in higher education (Farrington et al., 2012; Baars et al., 2015; Cromley et al., 2015; Kaplan and Patrick, 2016). These attributes include leadership, self-concept, self-set goals, attribution, and other skills related to student success. Because the MD attributes are connected to students’ outcomes, researchers and practitioners are using assessments to study how the MD attributes enable students to succeed in university settings (García, 2016).

Traditionally, self-report questionnaires and performance tasks are used to assess the MD attributes (Fernandez-Berrocal and Extremera, 2006; Duckworth and Yeager, 2015). The accuracy of the estimated ability with these traditional formats has been questioned in recent years for two reasons (National Academies of Sciences, Engineering, and Medicine, 2017). First, self-report questionnaires depend on the participant’s answer to one’s own behaviors, beliefs, or attitudes (Duckworth and Yeager, 2015; Crossley, 2016). As a result, items in self-report questionnaires can be misinterpreted by the participants, which can lead to inaccurate scores. Second, performance tasks rely on participants’ behavior under a situation that might not reflect a realistic situation (Duckworth and Yeager, 2015; Crossley, 2016). Consequently, the participants’ behavior in this format might not demonstrate how the MD attributes are manifested in their personal experience. Given traditional assessment formats might fail to obtain accurate scores for the MD attributes, the Motivational-Developmental Assessment (MDA) was developed in response to this concern.

Unlike the traditional assessment formats, the MDA consists of four open-ended writing prompts (Farrington et al., 2012; Kaplan and Patrick, 2016). Each writing prompt asks students to write about their life events, based on their personal experience. Trained raters score the students’ responses on the following six MD domains: self-awareness, self-authorship, coping, self-concept, self-set goals, and attributions of success or failure. Each writing prompt assesses three out of six MD attributes, but each skill is measured once in two prompts. Thus, the MDA format allows for a deeper understanding of how the students’ MD attributes are manifested in their personal experiences compared to more traditional rating scale formats. However, the format of the prompts may invoke a method effect in the form of a testlet effect.

### Method Effect

A method effect is unexplained variance beyond what is intended to be measured (Podsakoff et al., 2003, 2012). The method effect results from any aspect of a measurement process (e.g., different administration methods) and the test, including having a common measurement context, item context, or features of items (Podsakoff et al., 2003). As result, there are many types of method effects, including an order effect, a testlet effect, and rater effect (Maul, 2013). Once a method effect is identified in the data, it is possible to control for these effects, and avoid inaccurate parameter estimates (Podsakoff et al., 2012).

Different statistical approaches are developed to control for a method effect (e.g., Podsakoff et al., 2003, 2012; Maul, 2013). One way to model a method effect is using the confirmatory factor analysis (CFA) framework (Podsakoff et al., 2003). Conceptually, the method effect results in extra covariation not due to the assessed construct but reflects method effect variance. That is, method effect is an undesired latent variable not assessed by the test. Using the CFA framework, models (e.g., multi-trait multi-method CFA models) were developed, where a secondary latent variable could be estimated to control for a method effect (Podsakoff et al., 2003; Maul, 2013).

### Testlet Effect and Local Item Independence

A testlet effect is a type of method effect that occurs with the use of testlets (Maul, 2013). A testlet is a set of items clustered around the same stimuli (Wainer and Kiely, 1987; Rosenbaum, 1988). This effect results from violations of the local item independence (LII) assumption, a fundamental assumption related to classical test theory (CTT) and item response theory (IRT) (Yen, 1993; Schroeders et al., 2014; Raykov et al., 2015). This assumption is a prerequisite for IRT unidimensional models (de Ayala, 2009, p. 20) and has been the focus in simulation (e.g., Bradlow et al., 1999) and applied studies for IRT (e.g., Schroeders et al., 2014; Baldonado et al., 2015). For IRT, the LII assumption states that the probability of responding to an item relies only on the person’s ability to answer each question, and the item characteristics (Lord and Novick, 1968, p. 361). Thus, the probability of a response to an item does not influence the probability of responding to other items, controlling for ability. In the context of factor analysis, this assumption is known as the uncorrelated residuals assumption (Yen, 1993; Schroeders et al., 2014), where the indicator residuals are not correlated in the factor model. Overall, meeting the LII assumption indicates that item responses are solely explained by the latent variable(s) intended to be measured by the test.

Test formats might lead to violations of the LII assumption (Yen, 1993). One of these formats is the free-response format (Ferrara et al., 1997, 1999), such as with the MDA. We note that the MDA was not purposefully constructed with testlets. Responses are nested within the MDA’s prompts, similar to a testlet. In this situation, residuals within the testlets might be correlated. Consequently, local item dependence (LID) is present in the data, which can result in the testlet effect. In line with a method effect, the additional covariation between responses in a testlet might not be due to the measured construct but reflects testlet variance. Specifically, the testlet effect can be conceptualized as a secondary variable not measured by a test, but rather captured due to the administration of testlets. This secondary unmeasured variable might impact the performance of the examinee but could be accounted for in CFA models.

Ignoring the testlet effect in a scoring model can result in inaccurate parameter estimates (Koziol, 2016) and overestimating reliability estimates (DeMars, 2006). A unidimensional IRT model, for example, overestimates the discrimination parameter compared to a model that accounts for the testlet effect (Bradlow et al., 1999; Glas et al., 2000; Koziol, 2016). This overestimation can bias ability estimates and lead to possible incorrect decisions about the individual (Bradlow et al., 1999; Koziol, 2016). The inaccurate estimates are not exclusively tied to IRT. If models ignore the testlet effect, estimates can be inaccurate for factor analysis (Luo and Gordon-Wolf, 2019). Also, some reliability approaches based on CTT cannot be estimated in the presence of LID, such as the testlet effect (Raykov et al., 2015). To ensure test score accuracy in practice, the detection of LID is essential, and models should account for the testlet effect. Given this, bifactor models may assist with the detection of LID and modeling spuriously correlated responses. However, to date, this modeling strategy has been rarely used for such a purpose in educational and psychological research.

### Bifactor Models for the Testlet Effect

Traditionally, items are aggregated to create a single item (i.e., polytomous-item, super-item, or parcel) for each testlet (e.g., Hall et al., 1999; Bandalos and Finney, 2001). This super-item might control the effects of LID (or testlet effect) for the items within the bundle (Eckes, 2011). Next, these super-items are assessed via the unidimensional model. This model is used to support a scoring inference for test scores (e.g., Eckes, 2011). However, such practice ignores the items’ response pattern within the testlet. Given items within a group are aggregated to a super-item, parameters for each item (e.g., loadings) are not estimated (Wainer and Wang, 2000), but instead reflected in the super-item parameter estimates. Consequently, assessing each item’s quality in terms of item specific parameters might not be possible, and the estimated ability might not be accurate (e.g., low reliability, loss of information about the examinee’s ability). Given the above concerns, the testlet effect variable can be modeled as a specific factor(s) in CFA models (Maul, 2013). Though other models that account for the testlet effect are available (e.g., correlating residuals within a testlet, a second-order factor model; Braeken et al., 2007; Rijmen, 2010), we focus on two types of bifactor models (BM) to model the testlet effect, the conventional BM and the restricted BM.

**FIGURE 1:**
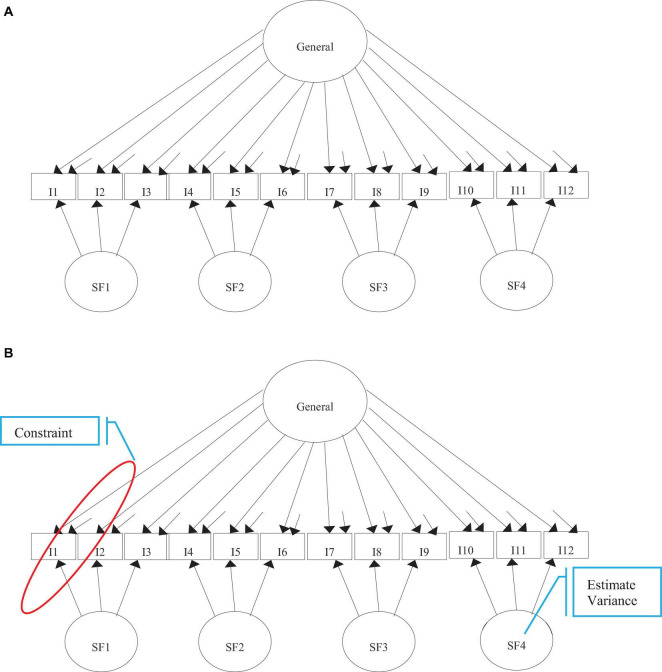
Path diagrams of the tested models. **(A)** Conventional bifactor. **(B)** Restricted bifactor. SF, Specific Factors.

Figure 1 presents the conventional BM and restricted BM. The conventional BM specifies a general factor related to all items to explain all the item responses (Gibbons and Hedeker, 1992), and a specific factor(s), which are related to certain sets of items within a testlet (DeMars, 2006; Hernandez-Camacho et al., 2017). Conceptually, the specific factors, not measured by the assessment, represents the testlet effect. Often, the factors are specified with a mean of 0 and a variance of 1 (e.g., Rijmen, 2010), and are orthogonal to each other (Gibbons and Hedeker, 1992). This specification results in two factor pattern coefficients for each item in the conventional BM, which are all freely estimated. The first pattern coefficient represents the relationship between the item and the general factor that is assessed. The second pattern coefficient represents the association between an item within a bundle and the corresponding specific factor(s) (Koziol, 2016; Hernandez-Camacho et al., 2017). The conventional BM takes the form (e.g., Muthén and Asparouhov, 2002):


(1) $$y_{i}^{*}=\nu +\Lambda_{y}\,\eta_{i}+\epsilon_{i}$$

where Λ_*y*_represents the pattern coefficients for the general factor and specific factor(s), and the ν is the intercept parameter. The η represents the vector for the general factor and specific factor(s). The ϵ represents a vector of the unique variance for each item that is not explained by the general and specific factor(s). The y* represents a continuous latent response variable for each observed categorical item i (Muthén and Asparouhov, 2002).

An alternative model to assess testlet data with LID is the restricted BM. The restricted BM, known as the testlet model, was originally developed from an IRT framework (Bradlow et al., 1999) and then connected to CFA (Luo, 2018). This model is proposed to detect LID and provide a scoring model that accounts for the testlet effect (Bradlow et al., 1999; Li et al., 2006). Like the conventional BM, all items are related to a general factor, and items within a testlet are associated with a specific factor(s), where again the factors are orthogonal.

In comparison to the conventional BM, the restricted BM requires two specifications in Equation (1). First, the general factor can be specified with a mean of 0 and variance of 1, whereas the specific factors are specified with a mean of 0 and random variance. The random variance on a specific factor provides the estimates for the random testlet effect parameter. This variance can quantify the magnitude of LID for each testlet (Bradlow et al., 1999). Second, each corresponding specific factor’s loadings are held equal to its respective loading on the general factor (Luo, 2018). Given these restrictions, this model is more parsimonious than the conventional BM (Hernandez-Camacho et al., 2017).

The conventional and restricted BM are connected in three ways. First, both models have the potential to assess testlet data that violate the LII assumption. Second, both models explain the item’s responses with a general factor and specific factor(s) for testlet data. Third, the restricted BM is nested within the conventional BM (Hernandez-Camacho et al., 2017). These models can assist with understanding the structure and scoring of the MDA and may be more promising in evaluating correlated responses than the unidimensional models with super-items (Hernandez-Camacho et al., 2017). Thus, the BM models have the potential to provide accurate estimates for the MDA scores while controlling for the testlet or method effect.

### Purpose

A single score is used for the MDA in practice (Kaplan and Patrick, 2016). This assumes that the one-factor model explains the MDA’s responses, and item’s responses within a writing prompt are independent. Given the MDA has a free-response format assessment, the MD attributes are nested within each writing prompt like a testlet. Thus, there can be the presence of a testlet effect for such data. In such a situation, ignoring the testlet effect can lead to imprecise parameter estimates for the MDA’s one factor model (e.g., Koziol, 2016). Thus, there is a need to evaluate the LII assumption with the MDA writing prompts. Given this, the BM models can be used to document the extent LID is present for each MDA writing prompt, model the one-factor MDA structure, and control the testlet effect, if indeed detected.

Given evidence is needed about the LII assumption and a scoring claim for the MDA scores, our purpose was to investigate a scoring inference for the MDA scores via BM models to detect LID and control for the testlet or method effect. A scoring inference informs how the MDA’s responses are related to the general factor (Kane, 2013), and how practitioners and researchers score the MDA. Two research questions (RQ) were addressed in this study.

RQ1: Is LID present in the MDA prompts as assessed with the restricted BM and other indices? We expect that the LII assumption will be violated, as detected by the restricted BM and Yen’s *Q3* index.

RQ2: Are responses for the MDA prompts explained by a unidimensional model, or by a unidimensional model that controls for the testlet effect? We investigated evidence to support a scoring inference for the MDA scores via factor analyses.

Results provide insight into the degree of support for the LII assumption and scoring claim for the MDA scores. The study also demonstrates how to detect LID and model correlated residuals via the conventional BM and restricted BM. The latter model is an innovative way to account for the testlet effect.

## Materials and Methods

### Sample and Procedure

The sample (*N* = 257; female = 75.4; 38.1% first generation) included first-year university students from a large research-focused university on the West coast of the United States. The assessment was completed in the first 4 weeks of the Fall 2018 semester. The assessment required approximately 10 min to complete.

### Instrument

The MDA (Kaplan and Patrick, 2016) consists of four self-reflecting writing prompts, which ask students to write brief paragraphs about their life events. Three out of six constructs are measured with each prompt. Hence, responses are nested within each writing prompt. Trained raters require approximately 10 min to score all prompts for each student. Each writing prompt for the MDA assesses different MD attributes. Prompt 1 assesses self-concept, self-set goals, and attributions of success. Prompt 2 assesses self-concept, attributions of failure, and coping. Prompt 3 assesses self-awareness, self-set goals, and self-authorship. Prompt 4 assesses self-awareness, coping, and self-authorship. The correlations between the six measured attributes ranged from 0.19 between self-authorship and self-concept to 0.73 between self-authorship and self-awareness, with a median correlation of 0.34 among all attributes.

### Raters and Rater Calibration

Three raters were trained and completed calibration in four sessions. First, raters were trained on the constructs and codebook by the MDA author. Second, raters practiced the ratings with examples. Third, raters met with the MDA author to discuss the inconsistencies in the practiced ratings and clarifying domain meaning. Finally, raters met consensus on scoring. Raters scored all responses over 10 weeks. To assess reliability of the MDA, we applied Generalizability Theory (G-theory). This allowed us to estimate reliability for the total score, and estimate variance associated with the object of measurement (i.e., the student), and variance associated with the raters and residual variance capturing error and other possible other facets not included in the design. G-theory analysis revealed that for MDA total score: (a) reliability (G-coefficient) was 0.91, (b) 72.8% of the variance was associated with the student, (c) 5.9% of the variance was explained by the rater, and (d) 21.3% variance was associated with the residuals.

Reliability values met the criteria to use scores for research purposes (i.e.,>0.80; Nunnally and Bernstein, 1994), and at least two raters (i.e.,>0.80) were needed to maintain adequate reliability levels. Tables 1 and 2 contain the descriptive statistics for the MDA total score and the six attributes scored across prompts, and for the individual attributes, respectively. As seen in Table 1, variability was similar across the attributes when scored across prompts. Attribution, self-authorship, and coping appeared to have the highest means out the six scores. Table 2 reveals that the attributes tended to be scored as present in prompt 1 and 2 at a higher rate compared to prompt 3 and 4.

**TABLE 1:** Descriptive statistics for the motivational and developmental (MD) total score and attribute scores across prompts.

MD scores	N	*M*	*SD*	Min	Max
Total score	257	5.64	3.26	0.00	12.00
Self-concept	257	0.85	0.73	0.00	2.00
Self-set goals	257	0.88	0.75	0.00	2.00
Attribution	257	1.28	0.78	0.00	2.00
Coping	257	0.99	0.74	0.00	2.00
Self-awareness	257	0.65	0.79	0.00	2.00
Self-authorship	257	1.00	0.87	0.00	2.00

*MD = Motivational and Developmental.*

**TABLE 2:** Percentage of students displaying an MD attribute by prompt.

Prompt	Attributes	N	Percentage
1	Self-concept	257	60.70
1	Self-set goals	257	49.42
1	Attribution	257	66.93
2	Self-concept	257	24.12
2	Attribution	257	60.70
2	Coping	257	47.47
3	Self-Awareness	257	26.07
3	Self-set goals	257	38.91
3	Self-authorship	257	52.14
4	Self-Awareness	257	38.52
4	Coping	257	51.36
4	Self-authorship	257	47.47

#### Analysis

For RQ1, the LII assumption was examined using the Yen’s *Q3* (Yen, 1984) via the *subscore* package (Dai et al., 2019) in the R statistical system and the restricted BM via using *Mplus* (Muthén and Muthén, 2017). We added the *Q3* to the detection of LID because it is a common index for LID detection and provide a confirmation check with the restricted BM results. *Q3* is the correlation between residuals (*d*) for item *i* and item *j*. *Q3* is expressed as:


(2) $$Q_{3}=r_{d_{i}\,d_{j}}$$

The detection of LID was conducted in two ways. First, following recommendations (Ferrara et al., 1997; Schroeders et al., 2014; Baldonado et al., 2015), we estimated the averages of the *Q3* values and the number of pairs with LID for each prompt. LID was determined to be present if *Q3* values ≥ 0.2 (Yen, 1984) for any three pairs of items in a prompt. Second, we estimated the variance of the testlet effect using the restricted BM. This variance identified LID for each writing prompt with the following criteria (Bradlow et al., 1999): 0.1–0.5 = low LID, 0.6–1.0 = moderate LID, and 1.1–1.5 = high LID. We recognize these criteria are guidelines and lack strong empirical support (e.g., Christensen et al., 2017). We still include these, given their use in practice, with a recognition that future work is needed to support these criteria.

For RQ2, a one-factor model, a conventional BM, and a restricted BM were examined for the MDA using *Mplus* (Muthén and Muthén, 2017), with the robust weighted least square estimator (WLSMV) to account for dichotomous data. Sample codes for estimating each model are provided in the supplementary material. The one-factor model ignored the testlet effect and served as a baseline to compare the BM models’ results. The conventional BM with four specific factors and the restricted BM with four specific factors were tested, following the specifications described in the introduction. These models were selected because they were alternative models to account for the testlet effect (e.g., DeMars, 2006; Li et al., 2006; Hernandez-Camacho et al., 2017).

### Model Evaluation

Model evaluation occurred in four steps. First, the following fit criteria were used (Brown, 2015): chi-square (χ^2^), comparative fit index (CFI ≥ 0.90), root mean squared error of approximation (RMSEA ≤ 0.05), and standardized root mean square residual (SRMR < 0.08). We apply these guidelines with caution, as ML-based fit indices have mixed support with robust estimators (Nye and Drasgow, 2011; Zhao, 2015; Xia and Yang, 2019; Shi et al., 2020). Second, the examination of parameters and standard errors provided information about the models’ performance, identified areas of misfit, and convergence issues. Third, the Δχ^2^ was employed to compare the conventional BM and restricted BM, given these models were nested (Hernandez-Camacho et al., 2017). A significant Δχ^2^ (*p <* 0.01) would indicate that the restricted BM fit worse compared to the conventional BM. Fourth, we examined the interpretability of the models in relation to theory. After identifying the best fitting model, internal consistency reliability was estimated via omega (ω) (McDonald, 1999). If the best model was one of the bifactor models, we also estimated omega hierarchical (ω_H_) to evaluate the variance attribute to the general factor (Rodriguez et al., 2016). Moreover, we tested additional models if modifications (i.e., reducing the number of specific factors) were justified with the conventional BM and restricted BM. These additional models were examined in an exploratory framework, as we did not have theory to specify the form of these additional models.

## Results

### Test for Local Item Dependence

Table 3 provides the estimated testlet effect variances and values for the restricted BM, and *Q3*, respectively. The testlet effect variance ranged from 0.21 to 4.08. The testlet effect variance for the restricted BM identified low LID for prompt 3 and 4, and high LID for prompt 1 and 2. The *Q3* averages ranged from −0.03 to 0.30 for items within the prompts. Further inspection of the *Q3* values identified three and two pairs with LID for prompt 1 and 2, respectively, and in agreement with the restricted BM results. Given agreement across methods, the MDA prompts 1 and 2 appear to violate the LII assumption. The one factor should not be considered for use, but its results are presented in tables and briefly mentioned in the next section for comparison and completeness purposes. Based on these findings, the MDA may need a scoring model that accounts for the testlet effect in prompt 1 and prompt 2.

**TABLE 3:** Summary of the presence LID for the MDA.

	Yen’s *Q3*		Restricted bifactor	
		
Prompt	*N*	*M*	σ_γ_^2^	Magnitude of LID
1	3	0.30	4.08	High
2	2	0.20	3.96	High
3	0	–0.04	0.20	Low
4	0	–0.01	0.21	Low

*σ_γ_^2^_=_ variance for specific factors. N = number of pairs identified with LID for each prompt. M = averages for Q3 values for each prompt.*

The multidimensional models were investigated in subsequent analyses to determine the best scoring model for the MDA. The results of RQ1 also guided the modification of the restricted BM and conventional BM, resulting in the reduced versions for these original models. Given that the magnitude of LID was small, reduced versions eliminated the secondary specific factor for prompt three and four.

### Modeling the Testlet Effect

Table 4 summarizes the fit for the one-factor, two original models (i.e., the conventional bifactor with four specific factors and the restricted bifactor with four specific factors) and two reduced models (i.e., conventional bifactor with two specific factors and the restricted bifactor with two specific factors). The original conventional BM failed to converge, resulting in an ill-defined solution. The one-factor model did not meet fit criteria. Model fit was not excellent for the reduced conventional BM, original restricted BM, and reduced restricted BM (i.e., all χ^2^ were significant, *p <* 0.05, CFI_*range*_ = 0.928–0.941; RMSEA_*range*_ = 0.100–0.113, SRMR_*range*_ = 0.117–0.121). That is, all fit indices did not meet set fit criteria. We note that even though these fit indices did not meet all fit guidelines, we did not continue to modify these specific models because we had no additional theoretical justification for modifications. Instead, we took an exploratory approach.

**TABLE 4:** Model fit indices for the models.

Model	χ^2^	*df*	*P*	CFI	SRMR	RMSEA	RMSEA-CI
One-factor	418.52	54	<0.001	0.835	0.180	0.162	0.148–0.177
Reduced conventional BM	205.69	48	<0.001	0.928	0.118	0.113	0.097–0.129
Restricted BM	179.43	50	<0.001	0.941	0.117	0.100	0.085–0.116
Reduced restricted BM (RRBM)	199.02	52	<0.001	0.933	0.121	0.105	0.090–0.121
Combined RRBM	89.79	53	<0.002	0.983	0.073	0.052	0.033–0.070

*CFI = the comparative fit index. RMSEA = root mean squared error of approximation. SRMR = standardized root mean square residual. CI = Confidence interval.*

We conducted a *post hoc* exploratory factor analysis (EFA). EFA has been used successfully to detect LID where item content and item location have been associated with LID (Stucky et al., 2011). Based on the scree plot and parallel analysis results, two factors were suggested. Inspection of the factor loadings suggested the first 6 indicators (prompt 1 and 2) and the last 6 indicators (prompt 3 and 4) formed factor 1 and factor 2, respectively. These factors had a low correlation (*r* = 0.36). These results were interesting, as they align with our results via the *Q3* and restricted bifactor model, suggesting that there is the presence LID for responses within the prompts, and prompt 1 and 2 have a stronger testlet effect compared to Prompt 3 and 4. Thus, the combination of the prompts into two factors could be indictive of locally dependent units representing a testlet or method effect, as seen in previous use of EFA for detecting LID (Stucky et al., 2011).

Given the above result, two additional *post hoc* CFA models were estimated with the same data. Both models allowed the items to be related to the general factor. First, a restricted bifactor model with two specific factors was specified using indicators from prompt 1 and 2 to form the first specific factor and indicators from prompt 3 and 4 to form the second specific factor. This model failed to converge. Second, a restricted bifactor model with one specific factor was specified using indicators from prompt 1 and 2 to form a specific factor. The fit of the latter model, the combined restricted reduced bifactor model (CRRBM), as seen in Table 4, met all fit criteria. Thus, the CRRBM was retained as the best fitting model for the MDA scores given it (a) was a parsimonious model in comparison to the other BM models, (b) accounted for a large presence of LID in prompt 1 and 2, and (c) was the only model to meet fit criteria. We did not engage in model comparisons via chi-square difference tests since the other models did not meet fit criteria and were not acceptable fitting model candidates.

Table 5 presents the standardized loadings for CRRBM, where loadings ranged from 0.19 to 0.92. The estimated omega (ω) was 0.93 for the total MDA score. Notice that this estimate, controlling for the method or testlet effect, was similar as the other bifactor models, as seen in Table 5. The ω_H_ was 0.69 for the scores from the CRRBM, accounting for the specific factor. Most of the reliable variance (0.74) in the total scores can be attributable to the general MDA factor, 24% is attributable to the testlet factor, and 7% is estimated to be random error. We also correlated factor scores of the general factor from the 5 tested models, which had a range of 0.96 to 0.99. That is, all models resulted in the same factor scores for the general factor. We recognize that more work is needed to continue to explore the underlying structure of the MDA, given our *post hoc* model fitting.

**TABLE 5:** Standardized factor loadings from the confirmatory factor analyses.

1-Factor		Reduced CBM			Reduced RBM			RBM					Combined RBM	
				
MDA	F1	F1	SP1	SP2	F1	SP1	SP2	F1	SP1	SP2	SP3	SP4	F1	SP12
Item1	0.640	0.372	0.826		0.373	0.822		0.401	0.810				0.306	0.834
Item2	0.518	0.284	0.736		0.315	0.694		0.337	0.682				0.253	0.689
Item3	0.623	0.383	0.744		0.356	0.783		0.382	0.771				0.287	0.782
Item4	0.292	0.162		0.701	0.240		0.524	0.259		0.516			0.186	0.508
Item5	0.425	0.355		0.461	0.297		0.648	0.323		0.642			0.213	0.580
Item6	0.402	0.322		0.763	0.329		0.720	0.352		0.700			0.203	0.554
Item7	0.759	0.787			0.788			0.750			0.335		0.796	
Item8	0.902	0.916			0.916			0.861			0.384		0.919	
Item9	0.903	0.920			0.921			0.865			0.386		0.925	
Item10	0.795	0.821			0.821			0.790				0.363	0.826	
Item11	0.810	0.838			0.837			0.792				0.364	0.844	
Item12	0.869	0.890			0.890			0.842				0.387	0.895	
ω	0.909	0.938			0.937			0.943					0.931	
ω_H_		0.794			0.797			0.768					0.688	

*F1 = General factor, SP = Specific factor corresponding to a prompt or prompts, ω = the omega coefficient for internal consistency reliability, and ω_H_ = the omega hierarchical coefficient for internal consistency reliability.*

*CBM = Conventional Bifactor; RBM = Restricted Bifactor.*

## Discussion

The purposes of this study were to evaluate the LII assumption and support a scoring inference for the MDA scores for use with university students through accounting for the testlet or method effect with a bifactor model. We focused on the MDA because it (a) is an innovative assessment, (b) contains responses that may violate the LII assumption, and (c) lacks evidence that examines this assumption and information to support a scoring inference. We also demonstrate how to apply a restricted BM to a non-achievement measure and the modeling of the testlet effect with the BM models. Collectively, our results demonstrate that the MDA’s prompt 1 and prompt 2 have the presence of LID, and the combined reduced restricted bifactor model supports a scoring inference for the MDA.

Results supported the presence of LID for the MDA through the restricted BM and the *Q3*. The magnitude of LID is high for the MDA’s prompt 1 and prompt 2 via the restricted BM. Complementing the restricted BM, *Q3* confirmed that LID exits for prompt 1 and prompt 2. The existence of LID in the MDA is aligned with the literature of free response format assessments (Ferrara et al., 1997, 1999). Our results suggest that LID appears most often in prompt 1 and 2 that ask participants to think and provide an explanation about an event where they feel success or failure and required self-evaluation about their abilities (self-concept). In Table 2, we also saw that more students displayed attributes in prompt 1 and 2 compared to prompt 3 and 4. It is possible that this higher rate of occurrence of these attributes, in these prompts is increasing the detection of the testlet effect in these prompts compared to prompts 3 and 4. A reviewer suggested that this could be a result of rater variance. However, G-theory suggests that variance associated with rater is low in this sample.

Results support a scoring claim demonstrating how the combined reduced restricted BM can account for the testlet effect. That is, to create factor scores for the MDA, one could use the CRRBM model, compared to the unidimensional model. A model that controls for the testlet or a method effect may produce better factor score estimates. That said, the testlet effect, at least in this MDA dataset, may not be strong enough to warrant the more complicated scoring model, as seen with the high correlations the factor scores (e.g.,>0.97) between the unidimensional model and the bifactor alternative models. The original restricted bifactor was not considered the best model for the MDA, as the presence of LID in two of the prompts was weak, and not likely to bias the parameters estimates (e.g., Koziol, 2016). The CRRBM allows all items to be related to one general construct and accounts for the testlet effect in prompt 1 and 2, where this effect is the strongest, by modeling this as one specific factor. Of course, this best fitting model was the result of exploratory and *post hoc* model specification and testing. This model would need to be replicated in a new and independent sample.

### Implications

Three primary implications for researchers and practitioners were identified. First, following the recommendations in the method effect literature (Podsakoff et al., 2003), we encourage future work to consider careful revisions for prompt 1 and 2 for the MDA to minimize the impact of the testlet effect. However, we acknowledged that revisions must be guided by the intended use of the MDA and underlying theory. It may be worth exploring the differences between prompts 1 and 2 and prompts 3 and 4 through techniques such as cognitive interviews, given the latter prompts assess overlapping constructs, but show small amount of LID and a lower attribute prevalence rate.

Second, we advocate the use of multiple statistics (i.e., restricted BM and other LID statistics) to detect LID for testlet data. Using the restricted BM and other LID statistics builds on the strengths of each approach to detect for LID. The LID statistic like *Q3* helps to detect specific pairs of items with LID without information about the factor structure of the MDA. However, the restricted BM provides the magnitude of LID for a testlet without detailed information about the item pairs, but it can help examine and support a factor structure for the MDA. The use of both approaches can provide additional information about the degree of LID and help ensure accurate detection of LID. This combination approach is like the use of multiple indices to judge model fit in SEM or the use of multiple criteria in evaluating factor models for invariance.

Third, we encouraged scholars and practitioners to incorporate the conventional BM and restricted BM into their research tool kit and apply these models as a control for method effects, such as the testlet effect, when appropriate. Such practice is an improvement over ignoring the testlet effect in a scoring model. Modeling the testlet effect can lead to accurate parameters and reliability estimates (e.g., Hernandez-Camacho et al., 2017). In fact, we saw that the omega estimates for the MDA scores when the method or testlet effect was controlled for was higher compared to the unidimensional factor model (e.g., 0.90 vs. 0.93). We acknowledge that estimation of models requires adequate sample sizes. Although the conventional BM can model the testlet effect, incorporating the additional parameters (e.g., two loadings for each item) requires a reasonable number of individuals to avoid convergence issues and produce accurate estimates (De la Torre and Hong, 2010; Jung et al., 2020). Thus, the sample size constraints would make the conventional BM approach less desirable to assess testlet data, compared to other approaches at this time. Given this, the restricted BM may assist with modeling testlet data. This may be helpful in applications where sample sizes are small, especially in non-achievement assessment programs (e.g., Mini-Mental State Examination; Rubright et al., 2016) and the MDA.

### Limitations

We identify several limitations of this study. First, the participants may not represent students from the diverse type of universities and regions in the U.S, given they were sampled from a single university located in a Western United States state. Other university students might have different experiences given contextual and cultural factors that may play a critical role in the degree to which MD attributes manifests within students, and hence the testlet effect could be different. In future studies, researchers should examine the scoring inference of the MDA among samples of students from other universities.

Second, measurement invariance of the MDA across groups was not considered. Such information informs if the factor structure, including the testlet effect of the MDA performs the same across different populations. Besides invariance, responses styles (e.g., Baird et al., 2017) could be an issue, as styles may also differ across student populations. In the absence of such evidence, the MDA scores may lack some precision, which requires additional work to understand. Future studies can investigate if the reduced restricted BM functions the same between groups, such as gender and ethnic groups. It may be the case that some interpretations of the prompts by students with certain backgrounds could yield less (or more) presence of a testlet effect for the MDA.

Third, our sample size was not large, and was based on a convenience sampling framework at a single public university. The sample size may have led to the convergence issues for the conventional BM with four specific factors, given large sample sizes are often needed to estimate the multidimensional models. The generalizability of our findings could be improved with a more diverse population of students drawn from more than one university.

Fourth, we used the standard cut-off criteria to identify LID via the restricted bifactor model and the *Q3* statistic in absence of other guidelines and strict thresholds for the *Q3* (e.g., Christensen et al., 2017). We acknowledge that the criteria (a) for the restricted bifactor model lack strong empirical support, and (b) for the *Q3* does not have specific thresholds. Given this, we opted to use the criteria defined by the Bradlow’ study for the restricted bifactor model (Bradlow et al., 1999) and commonly used thresholds in practice for *Q3*. Additional methodological work may help to understand how criteria function for these approaches under different conditions and with different types of assessments to accurately identify LID for testlet data.

Fifth, our modeling modification process was conducted in an exploratory framework. We did not have theory to guide additional model specifications. Instead, we used a two-step approach with exploratory factor analysis and CFA to identify a model that met fit criteria. However, given the novel application to account for the data structure we anticipated possible modifications would be suggested. Given the ex post facto modifications, we recognize that generalizability of CRRBM is questionable without validation or replication with independent samples. Our EFA results suggested that unidimensionality may not be supported, yet the identified two factors where indictive of testlet or methods effects as seen in previous work (Stucky et al., 2011). This was supported by the CRRBM, which fit well. Future work with the MDA will need to continue to explore the structure of the MDA.

## Conclusion

The MDA may assist in collecting a more in-depth understanding of students’ MD attributes compared to self-report questionnaires and performance tasks (Duckworth and Yeager, 2015). That said, the nature of such data might limit the usage of traditional scoring models, such as the unidimensional model, given the LII assumption can be violated. In this situation, the testlet effect, a type of method effect, can be present for the MDA’s writing prompts and must be controlled in a scoring model. In the CFA framework, other models (e.g., multi-trait multi-method) have been proposed to account for method effect where it is modeled as a specific factor (Podsakoff et al., 2003; Maul, 2013). Similar to such work, both conventional and restricted BM can control the testlet effect with specific factors, as we have demonstrated.

Both the conventional and restricted BM are alternative models that can be used with achievement and non-achievement measures. Our study demonstrates how to apply these models to non-achievement measures, as with the MDA. Beyond demonstrating how to control for this method effect with bifactor models, we provide valuable insight about the presence of LID within prompts and factor structure for the MDA, and likely other such measures. Given the call for more innovative measures beyond rating scales, this may be useful for other areas of assessment. Given the existence of LID, the CRRBM can be useful to provide accurate parameter estimates for the MDA. Additional validity evidence is needed to support the MDA score use among other university students and ethnic groups.

## Data Availability Statement

The original contributions presented in the study are included in the article/supplementary material, further inquiries can be directed to the corresponding author/s.

## Ethics Statement

The studies involving human participants were reviewed and approved by the Washington State University Institutional Review Board. The patients/participants provided their written informed consent to participate in this study.

## Author Contributions

DA was responsible for the main components of the writing and analysis. BF was responsible for assisting with the conceptualization of the idea and writing. Both authors contributed to the article and approved the submitted version.

## Conflict of Interest

The authors declare that the research was conducted in the absence of any commercial or financial relationships that could be construed as a potential conflict of interest. The handling editor declared a past co-authorship with one of the authors BF.

## Publisher’s Note

All claims expressed in this article are solely those of the authors and do not necessarily represent those of their affiliated organizations, or those of the publisher, the editors and the reviewers. Any product that may be evaluated in this article, or claim that may be made by its manufacturer, is not guaranteed or endorsed by the publisher.
